# Barriers and Enablers for Implementing Health Navigators in Paediatric and Adolescent Oncology in Chile: Local Insights From a Qualitative Study

**DOI:** 10.34172/ijhpm.9203

**Published:** 2026-08-18

**Authors:** Antonia Roberts, Francisca Vezzani, Carla Campaña, Báltica Cabieses, Alexandra Obach, Manuel Espinoza

**Affiliations:** ^1^Centro de Salud Global Intercultural (CeSGI) ICIM, Facultad de Medicina Clínica Alemana y Facultad de Psicología, Universidad del Desarrollo, Santiago, Chile.; ^2^Center for Cancer Prevention and Control (CECAN), FONDAP 152220002, ANID, Santiago, Chile.; ^3^Department of Health Sciences, University of York, York, UK.; ^4^School of Public Health, LKS Faculty of Medicine, University of Hong Kong, Hong Kong SAR, China.

**Keywords:** Paediatric and Adolescent Oncology, Health Navigators, Care Coordination, Chile

## Abstract

**Background:**

Childhood cancer poses significant public health challenges globally, with survival disparities between high-income countries (HICs) and low- and middle-income countries (LMICs). In response, the World Health Organization (WHO) launched the Cure*All* initiative to strengthen childhood cancer care. Despite being a HIC with a national oncology network, Chile faces fragmentation and unequal resource distribution similar to challenges in LMICs. The country recently introduced the role of *gestores oncológicos*, inspired by international health navigator models, to support patients and families through care coordination. This study describes the implementation of health navigators in paediatric and adolescent oncology in Chile from the perspective of healthcare professionals and health navigators, emphasising barriers and enablers to introduce this role in healthcare.

**Methods:**

A qualitative multiple case study was conducted through 30 semi-structured interviews with health navigators and healthcare professionals from public hospitals across Chile. Participants were purposively selected to capture diverse experiences. Interviews were analysed using content analysis, supported by triangulation and reflexivity to ensure rigour.

**Results:**

Barriers to implementation include structural issues, such as the lack of a national role definition and resource limitations, and team dynamics challenges linked to poor integration into clinical teams. Enablers included team openness to new roles and proactive strategies to introduce the navigator within the team, as well as individual qualities of navigators such as initiative, communication skills, and prior experience in paediatric oncology. Participants recommended that health navigators be full-time management roles focused exclusively on paediatrics. Key competencies include leadership, empathy, coordination, and organisational skills.

**Conclusion:**

Implementing health navigators aligns with the Cure*All* strategy by improving care coordination and system responsiveness in paediatric oncology. This experience in Chile highlights how barriers, particularly at the team dynamics level, can be addressed through communication and role clarity, offering valuable insights for other Latin American countries pursuing similar models of care.

## Background

**Key Messages**

**Implications for policy makers**

- This research emphasises the relevance of developing national guidelines to define and standardise care coordination roles across complex health services.
- This research highlights the importance of allocating sustainable funding for non-clinical support roles that improve care continuity.
- When introducing new roles in coordination and management in healthcare, it is relevant to promote multidisciplinary training programs that integrate clinical, psychosocial, and management skills for professionals who will perform these roles.
- This study shows the relevance of institutionalised onboarding and role-definition practices to ensure smooth integration of new roles into healthcare teams.

**Implications for the public**

When a child or teenager is diagnosed with cancer, families often face a complex and overwhelming health system. This research explored how a new role called “*gestores oncológicos*” is being implemented in Chile to support these families. Health navigators help guide patients through medical appointments, treatments, and referrals and provide emotional and practical support. The study found that this role can improve communication and reduce delays, contributing mainly in difficult to navigate systems. These findings are valuable not only for cancer care but for many areas where patients face similar challenges. By recognising and supporting roles that focus on care coordination, health systems can become more responsive, especially for vulnerable groups.

Childhood cancer constitutes a significant public health priority at the global level. According to Global Burden of Disease statistics, it constituted the second cause of death for the population under 20 years and the first cause for the group between five and 14 years in 2021.^1^ Studies have shown that children from high-income countries (HICs), referred to countries with an income of US $13 935 or higher in 2026,^2^ have good outcomes, with a survival rate of 80%. Still, children from low- and middle-income countries (LMICs), referred to countries with incomes between US $1135–$13 935,^2^ tend to survive less, with survival rates between 15% and 45%.^3^ These inequities reflect how the structure of societies affects population health, stating the relevance of intervening at the different levels of social determinants of health to improve childhood cancer. The health system plays a vital role at the intermediary level of social determinants of health because it can directly mediate the differential consequences of illness in people’s lives through access and can promote intersectoral strategies to improve health status.^4^ In the specific case of cancer in the paediatric and adolescent population, a disease that cannot be prevented or screened at current times, early detection and treatment are crucial,^5^ so interventions at the health system level related to diagnosis and treatment acquire particular relevance for improving health outcomes in this group.

 To address the global inequities in childhood cancer, the World Health Organization (WHO) launched in 2018 the Global Initiative of Childhood Cancer Cure*All*, aiming to increase prioritisation of childhood cancer in national policies and increase countries’ capacities to provide quality services for children with this disease.^3,5^ This initiative provides governments and stakeholders with a framework of key initiatives for childhood cancer care, including establishing specialised centres with skilled staff, integrating cancer services into universal health coverage, promoting context appropriate diagnosis and treatment, and strengthening monitoring and research.^5^ In this framework, WHO states that the possibility of delivering adequate healthcare to this group is directly related to the availability of trained human resources and that a lack of services and professionals, often identified in LMICs, can act as a bottleneck in the healthcare process, causing worse health outcomes.^5^ The Pan American Health Organization supports countries developing national childhood cancer plans in Latin America and the Caribbean (LAC). However, only a few countries in the region have specific plans for the paediatric and adolescent population.^6^

 The Chilean health system is mixed, with a significant public sector representing around 80% of the population and a private sector reaching a minor and more affluent portion.^7^ It is also segmented, with subcomponents of the health system directed to specific population groups, and fragmented due to the parcellation and specialisation of different levels and centres of attention.^8^ Although Chile is qualified as an HIC^9^ its health system faces challenges similar to those in LMICs, related to a lack of human resources in the public sector, disparities in the availability of professionals between urban and rural areas, and insufficient funding.^10^

 Regarding childhood cancer healthcare, Chile has an established and continuously progressing network, with 20 hospitals nationwide organised into low (support), intermediate (partial), and high (comprehensive) complexity centres. Five low complexity centres, located in five regions outside the capital, coordinate patient transfers for diagnosis and treatment, and provide maintenance treatment, palliative care, and follow-up. Four intermediate centres, also outside the capital, perform diagnostic tests, confirm suspected cases, administer oral treatments, and conduct follow-up checks. Eleven high complexity centres confirm diagnoses and deliver stage-specific treatments, carrying out all activities required for the oncological care of children. The network operates setting referral flows from lower complexity hospitals to more complex ones, which are mostly concentrated in the country’s capital.^6^ Of the 11 high complexity centres, six are located in the country’s capital.

 In terms of incidence of childhood cancer, Chile occupies the sixth place in the region, with an estimated rate of 14.2 per 100 000 children and adolescents. The most common cancer types are leukaemia, tumours of the central nervous system and non-Hodgkin’s lymphoma.^6^ Recently, the country developed a Childhood and Adolescent National Cancer Plan that incorporates a diagnosis of the national situation based on the Cure*All* initiative pillars and establishes the frames and aims to guide childhood cancer healthcare for the next five years.^6^ In terms of the diagnosis of the country’s situation related to centres and skilled staff, even though Chile counts with a functioning public network, challenges have been identified in terms of being adequately staffed according to the requirements for the care of children and adolescents with cancer. These challenges are similar to those faced by health systems in LMICs when addressing cancer.^5^ To address this deficit, professionals continue training themselves, and new roles are created. A new role recently created in oncology healthcare attention in Chile corresponds to *“gestores oncológicos,*” a term that has no translation to English but is based on thehealth navigators in oncology figures. This term will be used in the current paper. Health navigators are professionals from healthcare teams that aim to facilitate the trajectory through the healthcare system of the most vulnerable, marginalised and unattended patients, supporting the process of overcoming diverse structural, historical and profound barriers to access to healthcare.^11^ Internationally, three models of health navigators exist^11-13^: (*i*) one that classifies it as a cultural intermediary that has to acknowledge the community characteristics; (*ii*) one that states that they should focus on advising users on detection, diagnosis, treatment and survival, so they should have a professional clinical background; and (*iii*) one that suggests a multidisciplinary team of health navigators that considers community and clinical navigators working together. This role was initially created for adult oncology attention in Chile and later adapted to the paediatric and adolescent populations. It used attributes from the three existing models to define its characteristics.

 The role of health navigators in oncology is exclusively defined in the National Cancer Plan (Box 1).^14^

**Box 1. Definition of Health Navigators in the National Cancer Plan 2018-2028**

**Definition of Health Navigators in Oncology**

A designated member of the healthcare team who directly accompanies individuals diagnosed with cancer and their support network. They also manage, monitor, and coordinate the actions necessary to meet the objectives included in the treatment plan, manage support services, follow up on instructions and oversee the healthcare process for individuals.

Note: The Childhood and Adolescent Cancer Plan does not include an additional definition of the role, using the one previously presented.

This figure was established to support patients and families in basic navigation, identifying barriers and needs, delivering education regarding patient and family needs, coordination of care plans, referral and counter-referral management, and psychosocial support and assistance for support groups.^15^

 International evidence on the health navigator figure shows that patients value support for psychosocial needs and adaptations to overcome the diagnosis and treatment process,^16,17^ and that these programmes have positive results in terms of treatment adherence, cost-effectiveness, user satisfaction, and quality of life.^18-20^ Canada has been a pioneer in establishing clinical guides for standardising the role of health navigators, pursuing to guarantee quality in this practice.^21^ Still, most countries, like Chile, do not have specific guides for this role, which makes it challenging to integrate this role in all hospitals. Also, at the current times, there is no information related to how this has been implemented at the national level and if it contributes to improving coordination and overcoming fragmentation of healthcare in this population.

 Considering the relevance that interventions at the health system level can have in outcomes in paediatric and adolescent cancer, the novelty of the health navigator role in Chile, one of the few countries with specific paediatric and teenage cancer plan in the region, the characteristics of fragmentation and segmentation that increase the need to coordinate the attention in this health system, this study describes the implementation of health navigators in paediatric and adolescent oncology in Chile from the perspective of healthcare professionals and health navigators. This study emphasised on barriers and enablers to introduce this role in healthcare, aiming to provide evidence from a local experience that can inform other countries worldwide, with relevant data to implement improvements to reach the goals stated in the Cure*All* framework.

## Methods

###  Research Design

 This research was conceived from an interpretive paradigm, which comprehends reality as subjective and multiple.^22^ Methodologies that arise from interpretive paradigms help discern the meanings of experiences and can be used to inform and improve healthcare practices.^23^ This paradigm allowed us to delve into the experiences and meanings that participants allocate to implementing health navigators. The qualitative approach selected was a multiple case study, which consists of constructing a detailed understanding of the topic of study by gathering information from various cases.^24^ Each type of actor contemplated in this research was considered a case (health navigators and healthcare professionals), granting a deep understanding of the implementation experience.

###  Setting of the Study

 The study was conducted at the national level using online platforms. It considered eligible healthcare professionals who worked in the public sector from all institutions with oncology units nationwide. It focused on the public sector, serving the country’s largest population. Professionals from 12 institutions with oncology units distributed in different areas of the country participated. The study was carried out between March and November 2024.

###  Sampling and Recruitment

 The sample was purposive, and theoretical criteria were used to identify relevant profiles related to the topic of study.^25^ The researchers tried to represent the diversity of healthcare teams’ experiences implementing health navigators in paediatric and adolescent oncology healthcare. The following inclusion criteria were used: (*i*) healthcare professionals who worked in paediatric and adolescent oncology healthcare, (*ii*) health navigators who worked in paediatric and adolescent oncology healthcare. The following exclusion criteria were used: healthcare professionals who worked in paediatric and adolescent oncology healthcare for less than three years. This was stated so the participants could identify changes in healthcare by introducing the figure of health navigators.

 Recruitment was conducted through email invitations to each health navigator in the country and healthcare professionals in paediatric oncology units in each region. The emails were accessed through snowball sampling,^26^ starting with previous research team contacts. When recruiting, it was pointed out that participating in the research was voluntary and free of choice. None of the participants withdrew from the study during the interviews or afterwards. Characteristics of the participants are available in Table 1.

**Table 1:** Participants Characteristics

**Characteristics**			N
Gender	Female		28
	Male		2
Role in paediatric and adolescent oncology	Health navigators		9
	Health professionals	Oncologist	7
		Paediatrician	1
		Nurses	7
		Paediatric dentist	1
		Social worker	2
		Kinesiologist	1
		Psychologist	2
Region of the paediatric and adolescent oncology unit	North of the country		10
	Capital city		10
	South of the country		10

###  Data Collection

 Individual semi-structured interviews^27^ lasting 45 to 60 minutes were conducted online between April and August 2024, using the Zoom platform or WhatsApp video calls at convenient times for participants. This was favoured to reach participants from different regions of the country and to facilitate times when they could participate. Participants took part in the interviews from their homes or jobs. The researcher in charge of the interview ensured they took it from a space where they would be alone and feel comfortable.

 Interviews were conducted by AR, FV, and CC, all of whom had previous experience in qualitative research and interviewing. An interview guide was built around the study’s specific objectives, considering the topics of (*i*) barriers to the implementation experience of health navigators in paediatric and adolescent oncology, (*ii*) enablers to the implementation of health navigators in paediatric and adolescent oncology, (*iii*) contributions of health navigators in paediatric and adolescent oncology to healthcare, and (*iv*) recommendations for the implementation of healthcare navigators in paediatric and adolescent oncology from their experience. The semi-structured approach admitted emerging categories from the participants’ perspectives in each one of these topics. Interview guide is available in Supplementary file 1.

###  Analysis and Scientific Rigour

 The interviews were manually transcribed Verbatim to a text file. Two research team members reviewed each interview separately to ensure that the information in the text was the same as that in the audio files. The analysis was carried out in Atlas.ti software using the content analysis technique.^27^ Content analysis followed the categories in the interview script and incorporated new ones from the participants’ perspectives. The data analysis was carried out by AR, FV, and CC, who cross-checked the categories, and AO, BC, and ME performed a subsequent review. All Verbatim quotes included in this article were translated from Spanish by a native English speaker. Qualitative scientific rigour was achieved by: (*i*) Triangulation of participants, which was applied, cross-checking the findings from different participants in the study, (*ii*) Reflexivity, which was applied, taking notes from each researcher during interviews and analysis phase, later cross-checked, to complement and delve into the thematic contents.^27^

 The code tree based on the content analysis is contained in Figure.

**Figure:**
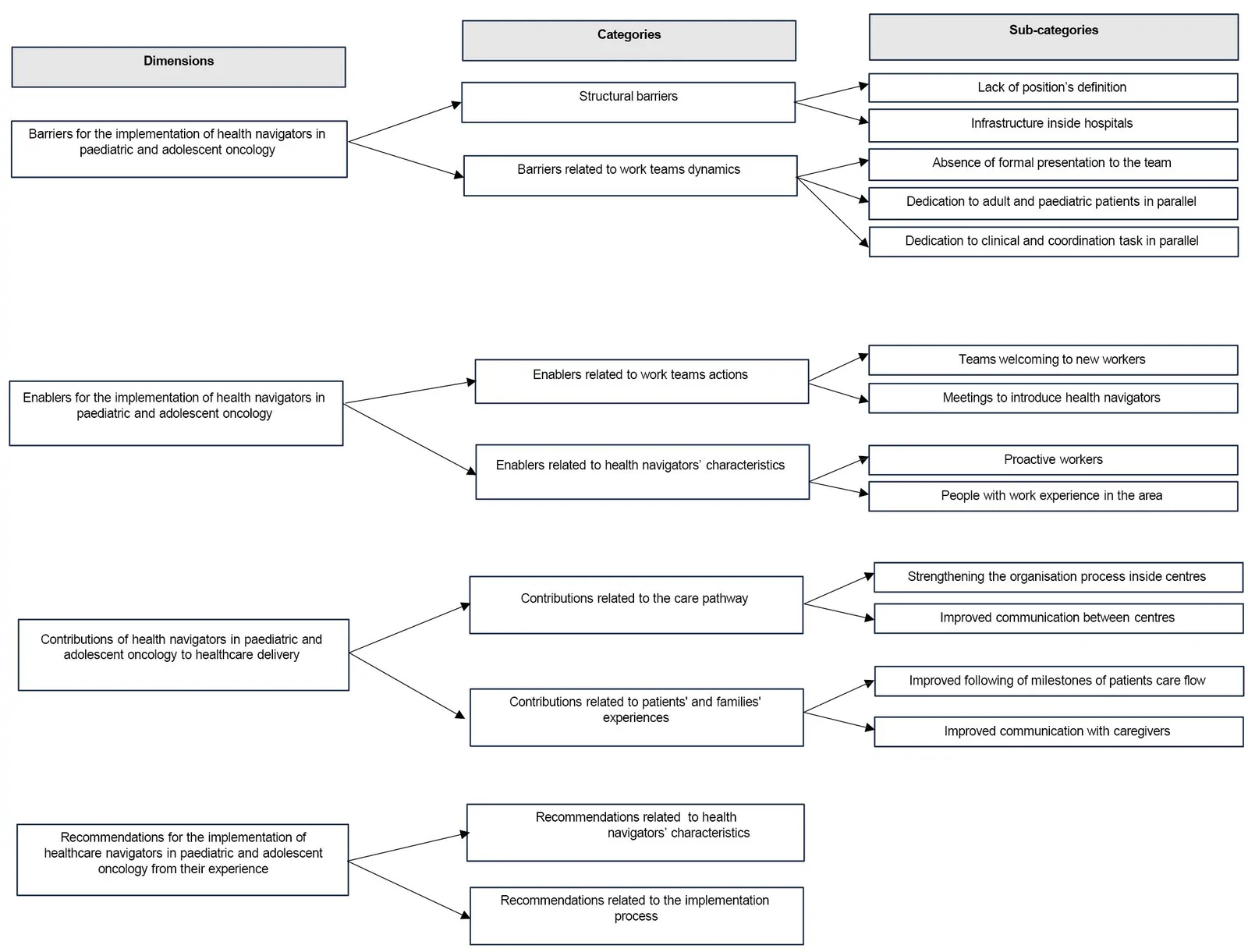
Code Tree.

## Results

 The results of the study are organised into four main sections, corresponding to: (*i*) barriers to the implementation of health navigators in paediatric and adolescent oncology, (*ii*) enablers to the implementation of health navigators in paediatric and adolescent oncology, (*iii*) contributions of health navigators in paediatric and adolescent oncology to healthcare, and (*iv*) recommendations for the implementation of healthcare navigators in paediatric and adolescent oncology.

###  Barriers to the Implementation of Health Navigators in Paediatric and Adolescent Oncology

 Participants identified various barriers to implementing the role within the teams. They have identified structural barriers that are linked to the position’s definition and the available hospital infrastructure. Also, barriers related to the dynamics inside the health teams were identified.

####  Structural Barriers 

 Regarding structural barriers, participants identified that the Ministry of Health defined the role of health navigators in general terms, establishing that the position would focus on accompanying families and facilitating the administrative processes required in the care flow. However, participants identified that the specific processes health navigators should manage were not specified. Considering this general approach, each hospital defined the exact tasks that this position should perform based on the local needs, human resources and physical space availability. This situation generated differences between hospitals, which participants relate to the geographical and complex characteristics of the health institutions. Consequently, the role was defined more expeditiously and specifically in the most complex hospitals where they receive a greater number of cases, all located in the country’s capital.

 “*The role, the tasks, the objectives or perhaps the mini objectives have had to be put together because, of course, the general objective is set out by the Ministry in the ordinance that describes our position, but it is like accompanying the patient, managing the things that the patient needs, so you have restricted resources that you have to prioritise and put together an order of what you are going to deliver, what you can provide, what is necessary to offer to the patients” *(Health navigator 4, country’s capital).

 Only in some hospitals, particularly the most complex ones, are health navigators’ part of the health service endowment. In others, the position is not officially assigned because the offer does not consider salaries adequate for the amount of experience required for a professional in this area, according to the participants’ perspectives. From the participants’ perspective, this relates to the fact that the management role in paediatric and adolescent oncology is not qualified as relevant due to a smaller number of cases compared to adult oncology and is left in second place compared to clinical needs. Due to the lack of resources inside institutions, opening a position in this area with adequate salaries is not prioritised.

 This situation is accentuated in less complex institutions, which receive fewer cases, perform fewer processes in the care flow and are concentrated in regions outside the capital.

 “*With all the profiles, everything, it could be perfectly open to competition [...] There is a tendency to minimise a little the work that is done, as care management, as coordination, as... not as relevant as what we see as a team, that is, more from outside than from within the team” *(Health professional 4, North of the country).

 Another structural barrier is the lack of infrastructure to introduce a health navigator in healthcare teams. This includes the lack of offices with desks, computers and telephones for management tasks and virtual infrastructure support, such as an email address for the health navigator to centralise processes. Also, the need for a space where they can provide support and guidance to family members in private was mentioned.

 “*And as internal problems that we have had is that the hospital has not yet managed, for example, to create a personal email for case management […] or a physical space because it does not have a physical space either, it is like it floats for the service, these types of difficulties we have faced in wanting to implement this post” *(Health professional 14, South of the country).

####  Teams Dynamics

 Regarding team dynamics, participants identified that health navigators started their functions in some hospitals without a formal presentation to the other healthcare professionals involved in paediatric and adolescent oncology. This acts as a barrier due to difficulties in establishing fluent articulation and identifying points where communication is needed between professionals.

 From the previous barrier, other barriers emerge related to different healthcare professionals’ unawareness of the health navigators’ role in paediatric and adolescent oncology attention. Due to the absence of a formal presentation to the team, which previously was in charge of duties that now are part of health navigators’ tasks, they feel that this new role is invading their functions, provoking conflicts inside teams. Consequently, the professional who assumes the health navigator role performs an auto-induction in some centres using hours of their schedule to coordinate meetings with different units to avoid or clarify conflicts related to the distribution of tasks.

 “*The first barrier was when the position came and everyone was like ‘What does this position do?’ Sometimes, they sent us things that didn’t correspond; sometimes, they didn’t send us certain information because they didn’t know our function. So, there was a lot of resistance to change where they didn’t know what these people who came to do, to invade” *(Health navigator 7, South of the country).

 Another barrier identified by participants emerges when health navigators in oncology units are not explicitly dedicated to the paediatric and adolescent population. This scenario manifests in hospitals that are not exclusively paediatric, where health navigators are principally focused on the adult population and functions related to paediatric and adolescent patients are added as an additional task. This situation makes it impossible for health navigators to prioritise tasks related to paediatric and adolescent patients due to the smaller number of patients compared to adults.

 Additionally, it is mentioned as a barrier that professionals dedicated to clinical tasks are destined to perform health navigator tasks in parallel, making it impossible for them to have protected schedules for coordination tasks. This prevents them from fully meeting the needs of the service in this respect and being trained in the required skills.

 “*Clearly, I have barriers, that is, I have to tell you that I don’t have exclusivity in this. Therefore, I have to take up time I don’t have for this to work, so I don’t have the protected timetables, right? No timetables would allow me to do it because I don’t have the position. Nor am I eligible for health navigator training, or training in the service, for someone to come and train me, because I haven’t had any training. I’ve only learned like ‘oh, what does the health navigator do?,’ asking around, watching webinars, and being there like ‘aha,’ but in reality, I don’t have, nobody has come to tell me ‘Look, you have the position of health navigator, this is what you have to do’” *(Health navigator 1, North of the country).

###  Enablers to the Implementation of Health Navigators in Paediatric and Adolescent Oncology

 Two types of enablers were identified from the participants’ perspectives. First, enablers were related to work team actions; second, they were related to health navigators’ characteristics.

####  Work Team Actions

 Concerning the teams’ actions, it is noted as an enabler that when the teams are open and welcoming to new people, despite the lack of knowledge about the health navigator role, they can be quickly introduced into the dynamics. In this regard, they highlight the holding of large meetings that enable the teams to get to know each other and to identify through this conversation the needs of each area of care and the processes in which the health navigator should be introduced in each case. This requires that the teams recognise that the health navigators’ role is necessary within the care. This positively influences the transfer of the required tasks and the coordination of the division of labour among the professionals involved.

 “*Our team as oncology referents in the health service are very close; they are very, very human people, very close, so from day one, we had meetings to get to know each other so that ideally we could work as well as possible. I think that was a very important point for us to be able to function and work smoothly in everything” *(Health navigator 8, South of the country).

####  Health Navigators’ Attributes

 About the characteristics of the health navigators, it is mentioned that they should be proactive in helping to install the role within the team by identifying tasks and flows and by establishing relationships with those who need to be involved in carrying out the position. Another enabler linked to the characteristics of the health navigators is that they should be people with work experience in the area so that they know the dynamics of patients and care within paediatric oncology. Thus, they enter with a knowledge base that facilitates the installation of the role within the teams.

 “*She has also had her work experience with paediatric patients, and she knows children who are patients, so she has a history, contact skills, and the ability to communicate with the different members of the team. I think that in the short time, we have seen a movement and articulation of the different instances” *(Health professional 15, South of the country).

###  Contributions of Health Navigators in Paediatric and Adolescent Oncology to Healthcare 

 Participants identified two types of contributions by health navigators: contributions related to the care pathway and contributions related to patients’ and families’ experiences.

####  Contributions Related to the Care Pathway

 The participants reiterated that the health navigators had enabled a better flow of care within the service, strengthening the organisation of the processes and freeing other clinical teams from coordination and accompanying areas. From their perspective, this is achieved by centralising the processes in a single person who can identify the path to be followed in their completion, which improves the flow from one stage to another. This way, the person can locate service needs at different stages and incorporate specific actions to escalate the identified needs to management.

 “*So it has freed up time for us to do patient summaries and clinical management rather than this other management part. So, it has been evident and a great contribution to the team” *(Health professional 16, South of the country).

 Additionally, it is perceived that communication between higher and lower complexity centres is better coordinated through health navigators. Centralised information can be easily shared with other institutions, reducing the time it takes to collect information on a patient. This speeds up processes in less complex institutions, such as initiating a patient’s follow-up stage.

####  Contributions Related to Patients’ and Families’ Experiences

 Improvements are seen in the timely fulfilment of their needs regarding examinations, transfers, and various care processes. This is achieved because the health navigator follows up on all milestones that must be met. In addition, they maintain communication with patients and remind them of relevant milestones, such as undergoing tests, so they do not get lost in the care process and/or manage to catch up with those who missed check-ups within the agreed periods.

 “*Yes, no, I mean, I have noticed it and it has been a clear benefit, the truth is that, as I said, before, I used to see patients without the MRI and without the report, and that has not happened to us anymore” *(Health professional 16, South of the country).

 In some hospitals in the capital city, the health navigators’ role is perceived as improving communication with caregivers. By centralising knowledge of the different processes, the health navigator can more fluently communicate to families the steps that are being carried out. In addition, in some centres, the health navigator has positioned himself as the person to whom caregivers turn to ask questions. In this respect, the teams perceive that they provide greater peace of mind to families.

 “*Now we have a person who is dedicated, with a focus, on these elements, so I feel that it was a great advance, and I also feel that it is a greater closeness, that the patients, who are calling them, is like seeing what they are doing and, therefore, a reassurance for them that maybe they are waiting a little bit for an exam, but there is someone seeing that and reassuring them that it will come out and that the whole study will be on time, so I thought it was very positive” *(Health professional 1, country’s capital).

 While this study focused on barriers and enablers for implementing health navigators at the national level rather than institutional comparisons, variations emerged across hospital complexity levels. To clarify these variations, Table 2 summarizes key differences along selected dimensions between High complexity centres (comprehensive, located in the country’s capital) and Intermediate/low complexity centres (partial/support centres, located in regions outside the capital). These centres were grouped due to the limited differentiation in participants’ accounts. This approach complements the previous analysis of barriers, enablers, and contributions across settings, illustrating implementation variability and the contextual factors shaping it.

**Table 2:** Comparative Summary of Health Navigator Implementation Variations Identified by Hospital Complexity Levels

**Implementation Dimension of Variation**	**High Complexity (Comprehensive Centres) **	**Intermediate/Low Complexity (Partial And Support Centres)**
Case volume	High number of cases	Lower number of cases
Role definition	Role had to be defined more expeditiously and specific	Role tends to be less specified
Position formalization	Tends to be a part of the official health service endowment	Tends to be not officially assigned
Infrastructure availability	Tends to be better equipped to introduce the role expeditiously	Tends to have significant gaps to introduce the role expeditiously

###  Recommendations for the Implementation of Healthcare Navigators in Paediatric and Adolescent Oncology 

 The participants made several recommendations regarding the knowledge and skills profile that health navigators should meet. They also outlined several administrative elements and team dynamics specifications that must be in place for health navigators to be adequately implemented.

####  Recommendations Related to Health Navigators’ Role and Characteristics

 It is suggested that the role should be explicitly established for management, not a position with half clinical and half management hours, as is currently the case in several facilities where the participants work. This would allow them to prioritise processes and avoid bottlenecks. At the same time, they indicate that the ideal is for it to be a position exclusively for the paediatric area, as sharing functions between adults and paediatrics does not allow the required tasks to be fully fulfilled. This requires specific hospital resources to open a position with these characteristics.

 Regarding health navigators’ characteristics, they should have detailed knowledge of the clinical field of oncology paediatrics and its programmes, protocols and norms. Ideally, they should have experience working in units of this type. This would enable them to understand the necessary procedures and timelines and to perform management tasks in coordination with clinical needs.

 “*It is very important that the person who is a case manager in paediatrics knows how to work in paediatric oncology. What role does everyone have, and what are the networks. In other words, it’s not just about receiving paperwork, phoning and asking for an appointment... It’s about knowing the networks where you are working and, above all, how the unit itself works” *(Health navigator 1, North of the country).

 It is recommended that health navigators should be health professionals, ideally nurses, as they have the necessary knowledge in clinical and management terms based on university training and the experience they acquire within paediatric oncology units. However, some participants point out that the role of case manager could also be developed by other professionals linked to care, eg, social workers or psychologists so that they know management processes and can relate more closely to the needs of patients and families.

 “*Maybe a social worker because you have to work a lot with other professions and manage things, maybe the social worker would still be useful” *(Health professional 21, country’s capital).

 A series of skills that health navigators should possess are set out. First, leadership skills and proactivity in managing work teams are highlighted. Communication and active listening skills are also required to liaise with the teams and the patient’s caregivers. Kindness is also needed to communicate information, particularly concerning caregivers and explaining complex processes. Also, they need to be empathetic and charismatic, understand the concerns of caregivers, and adequately accompany the processes, providing the information required by the families.

 “*We work directly with the caregiver or the child’s support network, so you have to have a very important soft skill to be able to handle certain cases because the parents or caregivers are obviously very anxious, in a stage of denial, they are angry with everyone, and you try to empathise and understand that their child is ill with a disease that may not be cured. So, we have to play a lot of a psychologist’s role to contain and learn to manage these situations that are suddenly a bit frustrating and stressful for us, which is challenging” *(Health navigator 8, South of the country).

 Another relevant skill is being meticulous and organised. This allows health navigators to keep an up-to-date record of all the processes carried out with a patient and those still pending to meet the defined timelines and detect possible bottlenecks in the process or delayed services.

####  Recommendations Related to the Implementation Process

 Participants suggested that there should be general guidelines at the national level that are clear about the specific functions of health navigators. Interviewees perceived that general delimitations were provided but not an in-depth description of the functions, which could be positive in standardising the position at the national level and facilitating its implementation within the services. To this end, the importance of drawing on the experience of both international and national hospitals implementing the health navigator role for longer, whether in the adult or paediatric area, is highlighted, as they can learn from both experiences how to implement the role more effectively.

 “*To be able to guide us according to the experience of older hospitals that have been implementing the role for some time, and well, this role is also in other countries, that is, not only here in Chile, in the United States they have been implementing the role of health navigator for years, so, of course... I imagine that this is to gather information, right, through this role in developed countries” *(Health navigator 3, North of the country).

 Another recommendation is to introduce the health navigators to the healthcare team formally. This can help avoid barriers related to miscommunication and role distribution inside units. Also, it is stated that they should be allowed to participate in all oncology committees so that they can know in detail the needs of patients and the characteristics of their health situation, thus facilitating the management required for each case. In addition, they need to be able to join clinical team meetings so that health navigators are directly immersed in the dynamics of patient care and needs, even though their position is mainly focused on management.

 It is also suggested that teams first identify the stages currently followed by patients in the care flow so that the processes are mapped out when a person is introduced to perform these tasks. Identifying the stages makes it possible to be transparent about the processes where the health navigator will be introduced and avoid duplication of functions between professionals. Standardising management and referral flows implies that management knowledge is not person-dependent and is available to teams broadly and transparently.

 “*I don’t know if one could perhaps specify their functions because, for example, when they explained to me what the function of the health navigator is, they are very general things, and it is like a position that has many things, very multidimensional, perhaps to be able to narrow it down and generate more explanatory guidelines so that one can know what the functions are” *(Health professional 18, country’s capital).

## Discussion

 This qualitative study aimed to describe barriers and enablers to introduce the role of health navigators in paediatric and adolescent oncology healthcare in Chile from the perspective of different health professionals. Even though this study may appear to delve into a particular policy in a specific area of healthcare from a small country, it can contribute multiple insights that can inform broader discussion about health systems in LAC and worldwide.

 Health navigators, commanded to facilitate the trajectory of patients in the healthcare system, particularly those who face the most barriers,^11^ play a central role in highly fragmented health systems like the Chilean,^8^ which is a shared characteristic of various health systems in LAC.^28^ The results identified several improvements to healthcare in paediatric and adolescent oncology related to the care flow inside hospitals, between different institutions, and communication between healthcare professionals and families. These improvements are coherent with the goals stated in the Childhood and Adolescent National Cancer Plan that aligns with the Cure*All *strategy, which aims to reduce bottlenecks in the healthcare process and increase the availability of healthcare professionals with adequate training for the required needs.^5^ Even though in this strategy the emphasis is made on clinical trained staff, the results of this study show the relevance that it has an adequate healthcare role of management that can improve processes and take the burden off other clinicians of coordinating tasks, so that they can have more time to devote to direct care. Thus, roles like this, introduced in complex flowcharts of attention that require multiple units to be coordinated, can also help make services more people-centred, making concrete moves to strengthen healthcare’s responsiveness, an intrinsic goal of health systems worldwide.^29^

 Despite the contribution of this figure, the study also identified several barriers to their implementation that also represent opportunities for intervention. Structural barriers like the absence of a standardised definition of specific roles at the national level and the lack of economic resources in some hospitals to open the position relate to major health system characteristics in Chile. The country has a mixed, fragmented and segmented system.^7,8^ Even though health expenditure has increased,^30^ it does not necessarily translate into efficient use or productivity in the health system.^31^ This difficulty in efficiently using resources is shared with other LAC countries.^32^ Regardless of the difficulty to intervene in structural characteristics of a country’s health system, recognising barriers that emerge in the clinical environment that specifically relate to structural issues can open spaces for discussion between clinical teams and decision makers based on concrete themes that both sides can comprehend, which can enrich the public debate and decision making on public health issues in the long term.

 In the short term, another set of barriers identified in the results related to the dynamics of the healthcare teams represents multiple opportunities for intervention that do not require economic resource relocation and are contained in the enablers found in some institutions. The leaders of the paediatric and adolescent oncology units can prevent difficulties in introducing this new role by ensuring formal instances for introducing the role to the units and defining the tasks that health navigators will perform. This enabler identified in the study aligns with literature that shows that effective communication between teams can act as an enabler for introducing interventions inside healthcare teams.^33^ This practice can strengthen teamwork dynamics in paediatric and adolescent oncology. It can also be introduced in other healthcare complex teams’ interventions, which helps address new evidence on a topic extensively studied that impacts the quality of care delivered to patients, staff fatigue, and burnout.^34,35^ Regarding the profile of health navigators, participants’ recommendations converged on various skills and knowledge they should manage related to fields like communication, coordinating work teams, and the clinical field of oncology. However, the results diverged in terms of professionals who should perform this role. Participants frequently recommended that nurses should be the adequate profession to perform health navigation tasks. Nevertheless, some participants suggested that other professionals, like social workers, might be better at fulfilling the health navigators’ tasks. Literature shows that the choice of who will be in charge of tasks related to care coordination depends on contextual factors and the population of interest, suggesting that social care expertise is significant for patients with functional decline. In contrast, nursing clinical expertise may be more relevant for patients with severe pathologies.^36^ The lack of consensus on the most suitable professional background for health navigator roles has significant implications. The absence of a defined profile hinders the development of standardised training programmes, as different backgrounds require distinct training emphases. Nurses need greater focus on psychosocial competencies and community resource navigation, while social workers require additional training in clinical oncology and healthcare navigation. This variability can lead to inconsistent skill sets, limit knowledge transfer between institutions, and affect patient outcomes. Evidence from other contexts similarly shows wide variation in navigator roles and competencies across professional backgrounds and institutional settings, resulting in mixed skills, difficulties in comparing positions, and unclear role boundaries.^13,37^ The results of this study cannot establish a more suitable profession for this role. Still, they can contribute to discussing the relevance of introducing multidisciplinary approaches in paediatric and adolescent cancer care processes. Without systematic evaluation of how professional backgrounds affect outcomes like care trajectories, treatment adherence, or family satisfaction, health systems lack evidence to guide strategic decisions about navigator workforce composition. This is particularly critical in paediatric and adolescent oncology, where complex clinical and psychosocial needs may require hybrid expertise beyond any single profession.

 Multidisciplinary care can be defined as an integrated team approach to healthcare in which medical and allied healthcare professionals work together and develop an individual treatment plan for each patient collaboratively.^38^ This approach benefits the decision-making processes in clinical care,^38-40^ but it is still circumscribed exclusively to healthcare-related professions and the clinical setting. Considering the study results, it might be relevant to introduce other disciplines, such as social workers in healthcare teams, who can contribute to a more holistic and truly biopsychosocial approach. Social workers in this area can delve into complex cancer-related issues that exceed the clinical setting, providing support and access to assistance in other needs.^41^

 This study has several strengths, as it aims to delve from a qualitative perspective into the implementation process of health navigators, a novel intervention that seeks to strengthen paediatric and adolescent oncology healthcare from the management of care perspective. The lessons from this Chilean study have direct applicability to LMICs, particularly in Latin America. Chile’s experience as one of the few LAC countries with a specific paediatric and adolescent cancer plan provides valuables insights for countries at similar stages of health system development. This is particularly important as it considers the perspectives of the professionals directly involved in the implementation. A recent document from the Andean Health Organisation analysed the situation in Bolivia, Colombia, Chile, Ecuador, and Perú in relation to the Cure*All* pillars, identifying weaknesses in referral and counter-referral networks, treatment follow-up, and monitoring of care.^42^ In response, strategic objectives were defined to promote timely cancer diagnosis and referral through training, communication and collaboration. The results of this research directly contribute to this effort by identifying effective strategies for implementing health navigators to strengthen referral networks, enhance follow-up, and improve team communication, while also highlighting potential shared barriers with other countries, such as limited resources and team dynamics challenges.

 Beyond the region, evidence from LMICs on the main determinants of delays in childhood cancer care shows that poor communication, multiple referrals, and unclear care pathways contribute to diagnostic and treatment delays.^43^ In this context, well-implemented health navigation programmes can help reduce these risks, providing actionable insights for diverse LMICs settings. Results and discussion can also broaden the discussion regarding teamwork in healthcare and approaches to care delivery.

 Still, this study has some limitations that must be taken into account. First, it considered information from informants from specific institutions in each zone with paediatric and adolescent oncology units, which may not represent the implementation experiences in all public institutions in the country. This might leave some relevant information related to the theme out of the results of this study. Second, all health navigators in the study were nurses, which may not represent implementation experiences in institutions where health navigators are another type of professionals. Third, it focused exclusively on the public sector, and even though this is the sector that reaches the most considerable percentage of the population, it might not represent the reality of the coordination processes in the private oncology sector.

 Future studies in this area should compare experiences from oncology institutions directed to adults and the paediatric population with health navigators and exclusively paediatric and adolescent oncologic institutions with health navigators to identify the specific challenges that the implementation of this figure faces in each context. In this line, it would be helpful to compare improvements in the oncologic care flow detected in institutions with the health navigators’ figure to the care flow in institutions without this role. Finally, future studies can also focus on institutions with health navigators that come from professions like social work or psychology to describe the characteristics of their role and identify potential improvements from a multidisciplinary approach to coordination.

## Conclusions

 The present study aimed to describe the implementation of health navigators in paediatric and adolescent oncology in Chile from the perspective of healthcare professionals and health navigators. Our study highlights barriers related to structural characteristics of health systems, barriers related to teamwork dynamics, and enablers that can be reproduced in other contexts. Also, showed improvements to the healthcare flow that a figure like health navigators can have. This local experience can inform interventions worldwide related to paediatric and adolescent oncology improvements in line with the Cure*All *strategy, reelevating the importance of intervention coordination and management processes to improve healthcare in general.

## Acknowledgements

 The authors thank everyone who contributed to the project and the Center for Cancer Prevention and Control (CECAN), FONDAP 152220002, ANID, Santiago, Chile.

## Disclosure of artificial intelligence (AI) use

 Not applicable.

## Ethical issues

 The study followed all ethical recommendations for health research with human beings.^44^ The analysis of risk-benefits related to the study is favourable because the potential risks identified in the interviews with health navigators and healthcare professionals in paediatric and adolescent oncology were minimal. The study contemplated digital informed consent with details of the study’s aims. Participants gave written and spoken consent to participate. The study was approved by the Ethics Committee of the Faculty of Medicine, Universidad del Desarrollo (code 2024-01).

## Conflicts of interest

 Authors declare that they have no conflicts of interest.

## Supplementary files


Supplementary file 1. Interview Guide.
