# Supplementary material for: Barriers and Enablers for Implementing Health Navigators in Paediatric and Adolescent Oncology in Chile: Local Insights From a Qualitative Study
Source: Int J Health Policy Manag. 2026 Aug 18;15:9203. doi: 10.34172/ijhpm.9203 (PMC13770232; doi:10.34172/ijhpm.9203)
Supplement: Supplementary file 1 [file ijhpm-15-9203-s001.pdf]

Article title: Barriers and Enablers for Implementing Health Navigators in Paediatric and Adolescent Oncology in Chile: Local Insights From a Qualitative Study

Journal name: International Journal of Health Policy and Management (IJHPM)

Authors' information: Antonia Roberts[1,2], Francisca Vezzani[1,2], Carla Campaña[1,2], Báltica Cabieses[1,3,2]*, Alexandra Obach[1,2], Manuel Espinoza[2,4]

[1]Centro de Salud Global Intercultural (CeSGI) ICIM, Facultad de Medicina Clínica Alemana y Facultad de Psicología, Universidad del Desarrollo, Santiago, Chile.

[2]Center for Cancer Prevention and Control (CECAN), FONDAP 152220002, ANID, Santiago, Chile.

[3]Department of Health Sciences, University of York, York, UK.

[4]School of Public Health, LKS Faculty of Medicine, University of Hong Kong, Hong Kong SAR, China.

***Correspondence to:** Báltica Cabieses; Email: bcabieses@udd.cl

Citation: Roberts A, Vezzani F, Campaña C, Cabieses B, Obach A, Espinoza M. Barriers and enablers for implementing health navigators in paediatric and adolescent oncology in Chile: local insights from a qualitative study. Int J Health Policy Manag. 2026;15:9203. doi:10.34172/ijhpm.9203

Supplementary file 1. Interview Guide

For the interviewer – please remember:

- *Apply this interview guide only after the participant has signed the digital informed consent form.*
- *Have a backup audio recorder available in case the primary device fails.*
- *Continuously observe the participant's emotional state, pausing the interview whenever necessary and checking whether they wish to continue participating.*
- *When starting the recording, reconfirm the participant's consent to participate so that it is recorded as evidence.*

Part I: General characterization

Sociodemographic characterization questions:

- Name:
- Age:
- Profession:
- Institution where you work:
- Health service where you work:
- Region where you live/work:
- (For health navigators only) ¿Are you a general oncological health navigator or exclusively a paediatric health navigator?

Part II: Interview

0) Introduction

- In your service, how many oncological health navigators are? What tasks do they perform?
- In your workplace, how is the role of the health navigator defined? What role do they play in practice?
- Do you have health navigators specifically dedicated to paediatric care, or only general health navigators?

i) Barriers to the implementation experience of health navigators in paediatric and adolescent oncology

- Can you identify specific challenges or barriers related to the introduction of this role in paediatric oncology care?
- What gaps do you identify between what has been implemented and what is proposed by the National Plan?

ii) Enablers to the implementation of health navigators in paediatric and adolescent oncology

- Can you identify specific enablers to the introduction of this role in paediatric oncology care?
- Can you provide information on collaboration and coordination among paediatric oncology centres at the regional and national levels?

iii) Contributions of health navigators in paediatric and adolescent oncology to healthcare

- What do you think are the main needs of paediatric cancer patients and their caregivers that could be addressed by health navigators?
- What improvements or contributions do you observe from this role in the service compared to how the service operated without a health navigator?

iv) Recommendations for the implementation of health navigators in paediatric and adolescent oncology from their experience

- What key characteristics should a paediatric oncological health navigator have?
- What specific skills or knowledge do you believe are essential for health navigators in this context?
- What improvements would you suggest in the implementation process of health navigators to optimize their impact on paediatric oncology care?

v) Closure

- Is there anything else you would like to add or highlight regarding the role of paediatric oncological health navigators?
